# Impact of unhealthy lifestyle on cardiorespiratory fitness and heart rate recovery of medical science students

**DOI:** 10.1186/s12889-020-09154-x

**Published:** 2020-06-26

**Authors:** Lampson M. Fan, Adam Collins, Li Geng, Jian-Mei Li

**Affiliations:** 1 Department of Cardiology, The Royal Wolverhampton NHS Trust, Wolverhampton, UK; 2 Faculty of Health and Medical Sciences, University of Surrey, Guildford, UK; 3 School of Biological Sciences, Harborne Building, University of Reading, Whiteknights, Reading, RG6 6AS UK

**Keywords:** Physical activity, Cardiorespiratory fitness, Heart rate recovery, Lifestyle, Medical science students

## Abstract

**Background:**

Medical science students represent valuable labour resources for better future medicine and medical technology. However, little attention was given to the health and well-being of these early career medical science professionals. The aim of this study is to investigate the impact of lifestyle components on cardiorespiratory fitness and heart rate recovery measured after moderate exercise in this population.

**Methods:**

Volunteers without documented medical condition were recruited randomly and continuously from the first-year medical science students during 2011–2014 at the University of Surrey, UK. Demographics and lifestyle components (the levels of smoking, alcohol intake, exercise, weekend outdoor activity and screen-time, daily sleep period, and self-assessment of fitness) were gathered through pre-exercise questionnaire. Cardiorespiratory fitness (VO_2_max) and heart rate recovery were determined using Åstrand–Rhyming submaximal cycle ergometry test. Data were analysed using SPSS version 25.

**Results:**

Among 614 volunteers, 124 had completed both lifestyle questionnaire and the fitness test and were included for this study. Within 124 participants (20.6 ± 4 years), 46.8% were male and 53.2% were female, 11.3% were overweight and 8.9% were underweight, 8.9% were current smokers and 33.1% consumed alcohol beyond the UK recommendation. There were 34.7% of participants admitted to have < 3 h/week of moderate physical activity assessed according to UK Government National Physical Activity Guidelines and physically not fit (feeling tiredness). Fitness test showed that VO_2_max distribution was inversely associated with heart rate recovery at 3 min and both values were significantly correlated with the levels of exercise, self-assessed fitness and BMI. Participants who had < 3 h/week exercise, or felt not fit or were overweight had significantly lower VO_2_max and heart rate recovery than their peers.

**Conclusion:**

One in three new medical science students were physically inactive along with compromised cardiorespiratory fitness and heart rate recovery, which put them at risk of cardiometabolic diseases. Promoting healthy lifestyle at the beginning of career is crucial in keeping medical science professionals healthy.

## Background

Recently, the number of university students in subjects affiliated to medicine or health care has risen markedly in particular for females [1]. People often assume that medical science education provides greater knowledge to students to keep them healthy and fit. In recent years, great effort has been given on the well-being and the quality of life to patients and other populations [2, 3] but few studies have assessed the well-being of young medical science professionals [4, 5]. At the early stage of their careers, new medical science students are more vulnerable to adopt unhealth lifestyle, which may be due to the challenge of living independently without parental constraints, changing eating behaviour, high academic demands with longer sedentary period at lectures and computer-screen and managing an active social life in the meantime [4]. A recent survey reported that in comparison to pre-university life, new university students struggled to find time for exercise in order to keep them physically fit [4]. Understanding the impact of unhealthy lifestyle components on cardiorespiratory fitness (CRF) and heart rate recovery of new medical science students is an important step to keep this young generation healthy. However, few studies had examined the health of medical science students relied on self-reported data [4, 5]. Little information is available based on objective physical activity measures and the association between these measures and the lifestyle components for these early career health professionals.

Physical activity, optimal body mass index (BMI), diet, and non-smoking are recommended lifestyle components for cardiovascular health [2, 6]. Physical inactivity has been recognised as one of the leading causes of pre-mature mortality [7] with an estimated health cost in UK around £0.8 billion per year [8]. Current UK government National Physical Activity Guidelines recommends at least 2.5 h/per week (or 30 min/day) of moderate intensity activity for adults (19–64 years old) [9]. Regular exercise during early adulthood provides vascular protection and lower disease burden in later life. On the other hand, poor fitness and low aerobic performance are often associated with a sedentary lifestyle, which is a well-described risk factor for the development of metabolic and cardiovascular disorders [10, 11].

Cardiorespiratory fitness (CRF) has been widely used to measure individual’s aerobic exercise performance and is a predicative marker for cardiovascular health [11–13]. The maximal rate of O_2_ consumption (VO_2_max) is a measure of CRF [14–16] . Low VO_2_max is also an independent predictor of cardiovascular mortality and morbidity [17, 18]. Heart rate recovery (HRR) after the cessation of physical exercise is another established prognostic indicator for cardiovascular events and all-cause mortality [19]. The objective of this study was to investigate the impact of modern lifestyles on cardiorespiratory fitness of young medical science students. To do this, we collected information of lifestyle components (levels of physical activity, BMI, smoking, alcohol use, daily sleep period and sedentariness) and examined their possible correlations with CRF and HRR measured individually in a cohort of 124 new medical science students. Information and findings from this study can be used for planning and developing a holistic approach to promote physical fitness of young adults at university and to improve their life expectancy after university.

## Methods

### Study design and ethics

This study was approved by the research ethics committee of the Faculty of Health and Medical Science at the University of Surrey, UK. Volunteers, both male and female, were recruited randomly and anonymously from the first-year medical science students who attended the human physiology practical classes during year 2011 to 2014 in the Faculty of Health and Medical Sciences, University of Surrey, UK. Prior to the study, volunteers were provided with the study information and consent documents. The inclusion criteria were first year undergraduate students between the ages of 18–35 without documented medical conditions. Exclusion criteria were above the age of 35 and evidence of medical conditions. Participants who failed to answer all questions in the survey form or failed to complete the fitness test or missing exercise data recording were excluded from the study. Data were collected anonymously and entered electronically for statistical analysis.

### Demographics and lifestyle information and cardiorespiratory fitness assessment

Information pertaining to individual demographics and lifestyle components were collected through pre-exercise questionnaires specifically designed in whole for this study (Supplemental Additional file 1). Demographics included gender, age, ethnic origin, medical conditions and home country of residence. Lifestyle components included alcohol consumption (units/week); current smoking status (cigarettes/week); levels and duration of physical activity (in terms of exercise and sport, h/week); sleep duration (h/day); weekend outdoor activity (h/day), and sedentary screen-time including computing, watching TV and gaming (h/day), and self-perceptions of physical fitness. Current smokers are defined as individual who has smoked 100 cigarettes previously and currently smoke according to Centre for Disease Control and Prevention (CDC, USA) [20]. Body height and weight were measured and recorded by the investigators before the exercise test, and used to calculate BMI. Resting blood pressure (BP) and heart rate (HR) were taken prior to the fitness test. Åstrand–Rhyming submaximal cycle ergometry test was performed using the protocol as described previously [21, 22]. It consisted of a 6 min exercise bout on a cycle ergometer (KORR Medical Technologies Int, USA). Power output of 75–125 W was used for female, and 100–150 W for male. Participants were instructed to maintain a pedal frequency of 50 rpm. Heart rate during exercise was recorded continuously using an infra-red ear lobe clip monitor. The criteria for exhaustion were heart rate at ~ 185 beats per minute and a subjective judgment by the investigators that the participant could no longer keep up. CRF was expressed as V̇O_2_max (ml^.^kg^-1.^min^− 1^) calculated using the Astrand and Ryhming nomogram [22]. The heart rate recovery and the BP were measured for 3 min after the exercise while the participant was still sitting on the bicycle.

### Statistical analysis

Descriptive statistics included mean with standard deviation for continuous variables and frequency with percentage for categorical variables. If asymmetric then median with interquartile range were reported. A liner regression and hierarchical multiple regression analysis model was used to generate various regression equations for predicting the relative contribution of gender, BMI, PA levels and self-assessment of fitness with VO_2_max values or HRR at 3 min as the dependent variables. Pearson correlation and values at 95% confidence intervals (CI) were used to measures the statistical relationship (or association) between two variables of VO_2_max and HRR. Levene’s Test was used to determine the equality or homogeneity of variance of the groups. Statistical significance was set at alpha *P* < 0.05. Data analyses was performed using the Statistical Package for the Social Sciences (SPSS) version 25 software for windows.

## Results

### Demographics and lifestyle components linked to cardiovascular health

The information of demographics and lifestyle components were given in Table 1. There were 58 (47.2%) male and 66 (52.8%) females with an average age of 20.6 ± 3.7 years. There were 56.5% EU Caucasians, 12.9% Asian and 9.7% Africans and the rests were other ethnical origins. The majority (80.5%) of the students had BMI within the normal range (18.5–24.9 kg/m^2^), 11.3% were overweight (BMI > 25 kg/m^2^) and 8.9% were underweight (BMI < 18 kg/m^2^). Among the participants, 8.9% described themselves as current smokers. Approximately 33.1% of the participants admitted to consume alcohol ≥12 units/week (112 g/week), which is close to the UK safe limit of alcohol consumption (≤14 units/week or 112 g/week) [23] with males occupying the greatest proportion of this category.

**Table 1:** Demographics and lifestyle information of participants

	Male	Female	Total
Participants	58 (47.2%)	66 (52.8%))	124
Age (years)	20.7 ± 3.1	20.5 ± 4.1	20.6 ± 3.7
Ethnic origin			
Caucasian (EU)	36 (62.1%)	34 (51.5%)	70 (56.5%)
Asian	5 (8.6%)	11 (16.7%)	16 (12.9%)
African	6 (10.3%)	6 (9.1%)	12 (9.7%)
Others	11 (19.0%)	15 (22.7%)	26 (21.0%)
Residence			
UK	34 (58.6%)	37 (56.1%)	71 (57.3%)
EU	9 (15.5%)	13 (19.7%)	22 (17.7%)
Overseas	15 (25.9%)	16 (24.2%)	31 (25.0%)
Hight (cm, measured)	178.3 ± 6.3	165.6 ± 5.8	171 ± 8.8
Weight (kg, measured)	73.2 ± 9.8	59.7 ± 8.7	66.0 ± 11.4
Average BMI (kg/m^2^)	23 ± 2.6	21.7 ± 3.2	22.4 ± 3.0
Overweight (> 25 kg/m^2^)	8 (13.8%)	6 (9.1%)	14 (11.3%)
Underweight (< 18.5 kg/m^2^)	5 (8.6%)	6 (9.1%)	11 (8.9%)
Normal weight (18.5–24.9 kg/m^2^)	45 (77.6%)	54 (81.8%)	99 (80.5%)
Current Smoker	5 (8.6%)	6 (9.1%)	11 (8.9%)
Alcohol use			
≥ 12 units/week	26 (44.8%)	15 (22.7%)	41 (33.1%)
< 4 unit/week or no alcohol	32 (55.2%)	51 (77.3%)	83 (57.9%)
Moderate Exercise (h/week)			
≥ 3 h/week	40 (69.0%)	41 (62.1%)	81 (65.3%)
< 2.5 h/week	18 (31.0%)	25 (37.9%)	43 (34.7%)
Outdoor activity at weekend			
> 4 h/per week	28 (48.3%)	38 (57.6%)	66 (53.2%)
0-4 h/per week	30 (51.7%)	28 (42.4%)	58 (46.8%)
Screen-time at weekend			
> 3 h/day	44 (75.9%)	59 (89.4%)	103 (83.1%)
0-3 h/day	14 (24.1%)	7 (10.6%)	21 (16.9%)
Daily sleep			
< 7 h/day	35 (60.3%)	38 (57.6%)	73 (58.9%)
> 7 h/day	23 (39.7%)	28 (42.4%)	51 (41.1%)
Self-assessment of fitness			
Not fit	17 (29.3%)	26 (39.4%)	43 (34.7%)
fit	41 (70.7%)	40 (60.6%)	81 (65.3%)

Self-reported physical activity (any form of exercise or sport) (h/week) were evaluated according to UK National Physical Activity Guidelines for adults [9]. Of the 124 participants (Table 1), 65.3% had about ≥3 h/week of moderate exercise, 34.7% had < 3 h/week exercise and 5.6% did not engage in any form of exercise or sport at all. When asked for reasons for not meeting National Physical Activity Guidelines, the majority responded “no time”. When asked about outdoor activity at weekend, 53.2% students claimed to have in average of ≥4 h outdoors/per weekend and the rest did not. In contrast, 83.1% of students admitted to spend > 3 h of screen time /per weekend-day to play games, watch film or TV. Regarding the sleeping time, 58.9% of students admitted to sleep < 7 h/day which is below the recommendation by UK National Health Serves [24]. For the self-assessment of physical fitness, 34.7% of participants perceived themselves as being physically unfit (feeling tired) with females being the majority in this category.

### VO_2_max distribution, HRR and their associations with lifestyle components

**Fig. 1:**
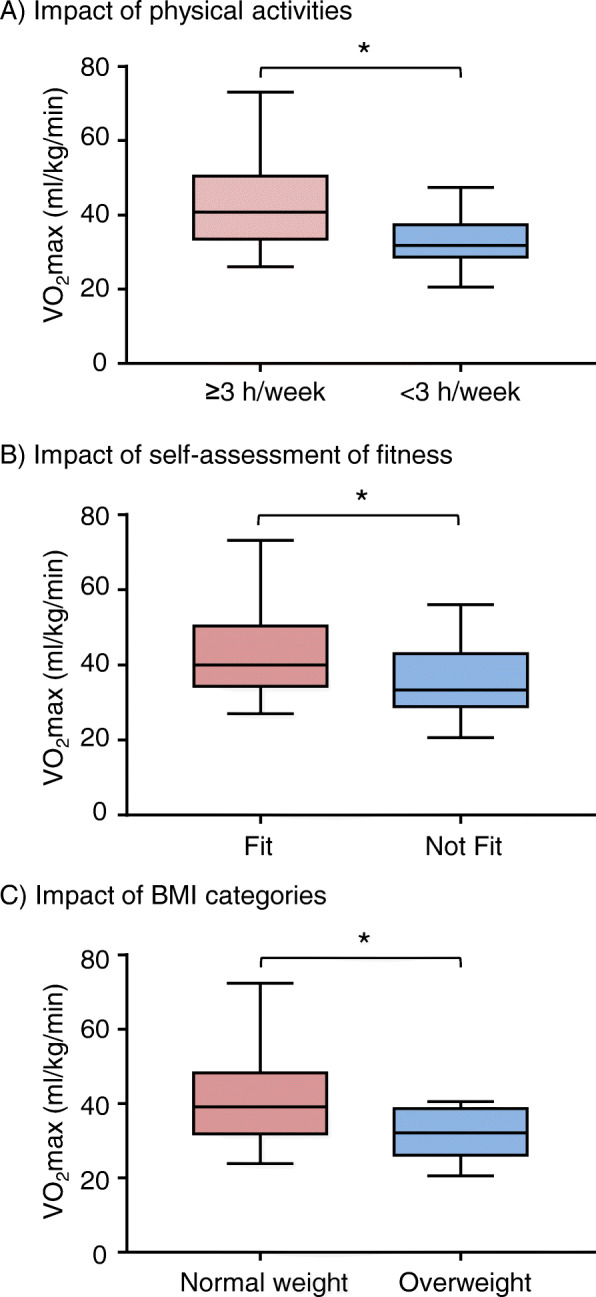
The impacts of the levels of physical activity, self-assessed fitness and BMI on VO_2_max distribution. **a** Differences in VO_2_max distribution between participants who had ≥3 h/week physical activity versus those who did not, *P < 0.0001. **b**. Difference in VO_2_max distribution between participants who self-precepted themselves to be physically fit versus those who felt not fit, *P = 0.001. **c** Differences in VO_2_max distribution between normal weight versus overweight participants. *P = 0.012

Significant correlations were found between the VO_2_max distribution and the levels of physical activity, self-assessed fitness and BMI (Fig. 1). Those who participated ≥3 h/week of exercise had significant higher values of VO_2_max distribution than others (F = 14.161; *P* < 0.001). The participants who believed themselves physically fit had significantly higher values of VO_2_max distribution than those who felt not fit (F = 11.43; *P* = 0.001). Overweight and obese group had lower values of VO_2_max distribution in comparison to the values of normal weight group (F = 4.555; *P* = 0.01).

**Fig. 2:**
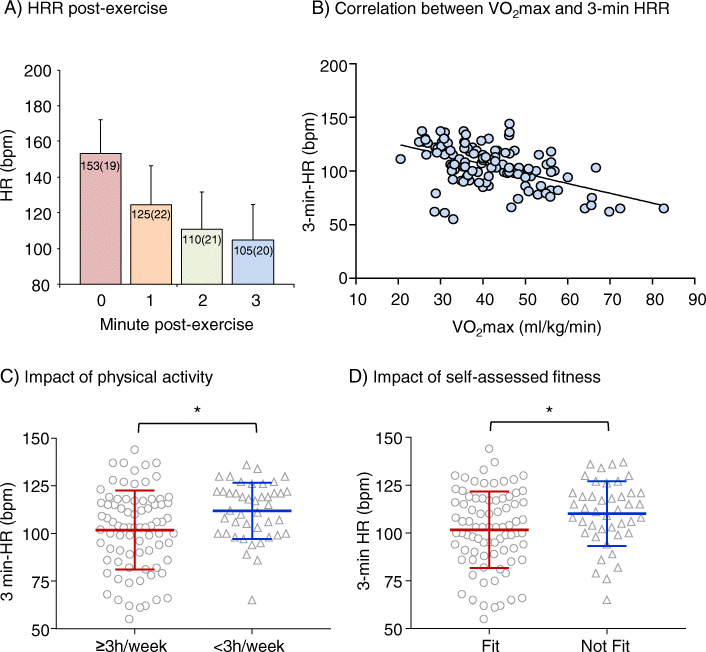
Heart rate recovery post exercise and significances. **a** HR measured every min for 3 min post-exercise. Values in the bar represent mean HR and (SD). bpm: beat per minute. **b** The correlation between 3-min HRR and VO2max. r = 0.673, *P* < 0.0001. **c** Impact of the levels of exercise on the values of 3-min HRR, *P = 0.005. **d** Impact of self-assessed fitness on the values of 3-min HRR. *P = 0.01

The HRR was measured for 3 min after the cessation of physical exercise (at time 0) (Fig. 2). The HR decreased quickly in the first min (> 25 beats/min), and then slowed down (Fig. 2a). At 3 min post exercise, the mean HR (105 ± 20) was still above the resting HR (80.8 ± 16.6) measured before exercise. We found that HR at 3 min post-exercise had a good correlation with VO_2_max distribution (Pearson correlation coefficient = 0.673; 95% CI: 0.646–1.175; *p* < 0.0001) (Fig. 2b). Participants who had < 3 h/week exercise had significantly slower HRR recovery at 3 min than others (F = 8.297; *P* = 0.005). Participants who believed themselves physically not fit had an attenuated HRR at 3 min in comparison to those who felt physically fit (F = 6.767; *P* = 0.01). Although approximately 33.1% of the new medical science students admitted to consume alcohol beyond the safe limit, we did not find significant association between the amount of alcohol consumption and VO_2_max (or HRR) in this study. We did not find association between daily sleep time with CRF and HRR.

**Fig. 3:**
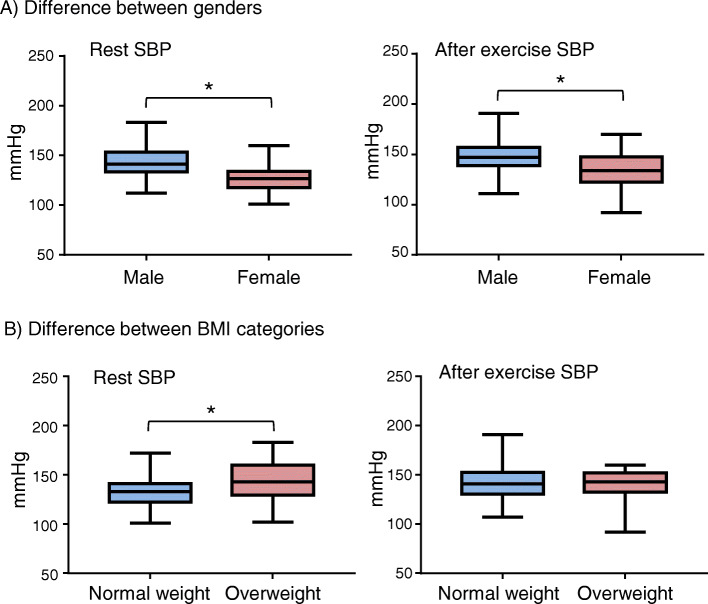
Impacts of gender and BMI on systolic blood pressure (SBP) at rest (left panels) and after exercise (right panels). **a** Differences in SBP between males and females. *P < 0.0001. **b** Differences in SBP between normal weight and overweight. *P < 0.01

We also found gender-influence on SBP that females had slightly but significantly lower SBP than males at both rest and after exercise (F = 42.121; *P* < 0.0001) (Fig. 3a). There was no significant difference for diastolic BP. BMI also had an impact on resting SBP. The overweight and obese group had higher resting SBP than the normal weight group (F = 4.934; *P* = 0.009) and this difference disappeared after exercise (Fig. 3b), suggesting exercise is beneficial for the young overweigh adults to prevent pre-hypertension syndrome.

## Discussion

Medical science students at university are valuable workplace resources for the development of new medicine and medical technology for a better healthcare. However, studying biomedical science does not mean the students will adapt a healthy lifestyle [4]. In the present study, we examined the lifestyle components through self-filled questionnaires and measured the CRF (VO_2_max) and HRR distributions of 124 new medical science students. We discovered that a significant proportion (34%) of these new students failed to meet the UK National Physical Activity Guidelines with CRF distribution and their 3 min-HRR values significantly below the values of their peers. Unhealth lifestyle behaviours together with lack of physical activity had put these young medical science students under greater risk of developing cardiometabolic diseases.

It is well established that low CRF in the young is a prominent risk factor for the development of cardiovascular disease, and regular exercise improves CRF and reduces mental stress [13, 25]. In accordance with previous studies, we found in the present study that VO_2_max distribution was positively associated with the levels of physical activity, self-perception of fitness and BMI. Overall, one in three biomedical students failed in keeping a healthy lifestyle profile of exercise according to UK National Physical Activity Guidelines and felt tired easily. Despite their education in medical science, these young adults had lower CRF and HRR values and increased risks for cardiovascular diseases at later ages.

Attenuated HRR is associated with increased risk of cardiovascular events and all-cause mortality [19]. However, previous studies referred HRR within 1 min or 2 min after exercises and were for hospital patients [19, 26]. A significant finding in the current study is a close relationship between the 3-min HRR with VO_2_max distribution and the levels of exercise and self-assessment of fitness. Further studies are needed to establish the clinical significance of 3 min HRR in predicting risk of cardiometabolic diseases in the general population.

It had been reported that bouts of exercise at least once during the weekend reduced the risk of metabolic and cardiovascular diseases [27]. Notably, the population in this study was age-matched young health professionals who spent their most weekday time sedentary for academic studies. However, when questioned about leisure-time outdoor activity during the weekends, nearly half of participants admitted to spend < 4 h outdoors of any kind of physical activities per weekend and the majority (83.1%) of students admitted to spending > 3 h/per weekend-day for sedentary screen time. BMI is inversely correlated with CRF which is in accordance with previous studies [27].

There are limitations because the study was conducted in one institution. However, the participants in our study were recruited continually over 4-year period containing a variety of ethnic origins with their home residence crossing several counties, and data were representative for this population. Another limitation is the lifestyle components and levels of habitual physical activity (h/week) were self-assessed. However, the VO_2_max and HRR of each participant were measured and their correlations to gender, BMI, PA levels and self-assessment of fitness were confirmed statistically.

## Conclusion

This study provides evidence that unhealthy lifestyle has a significant negative impact on the CRF and HRR of young medical science students. One in three participants failed to meet UK National Physical Activity Guidelines and self-perceived to be physically not fit along with their CRF and HRR values being significantly lower than the values expected for their age, which put them at increased risk of cardiometabolic diseases at later age. We found that 3 h/week exercise (any form and strength) at university is necessary for maintaining healthy CRF and HRR. We also found a good correlation between 3 min-HRR post-submaximal exercise with VO_2_max and the levels of physical activity, and 3 min-HRR can be a valuable indicator for cardiorespiratory function. Encouraging regular exercise and to implement healthy lifestyle changes should be a priority for the long-term health of young adults in medical science education.

## Supplementary information

**Additional file 1.** Activity & lifestyle questionnaire.

## Data Availability

The datasets generated during and/or analysed during the current study are available from the corresponding author on reasonable request.

## Abbreviations

CRF: Cardiorespiratory fitness
HRR: Heart rate recovery
BMI: Body mass index
