# Supplementary material for: Impact of unhealthy lifestyle on cardiorespiratory fitness and heart rate recovery of medical science students
Source: BMC Public Health. 2020 Jun 26;20:1012. doi: 10.1186/s12889-020-09154-x (PMC7318519; doi:10.1186/s12889-020-09154-x)
Supplement: Supplementary file 1 — Additional file 1. Activity & lifestyle questionnaire. [file 12889_2020_9154_MOESM1_ESM.pdf]

ACTIVITY & LIFESTYLE QUESTIONNAIRE

Tick the box or circle the right answer

Age _____ Gender: Male ☐ Female ☐

Height ______cm Weight ______kg

Ethnic origin: Asia ☐ Africa ☐ Caucasian (EU) ☐ Other ☐

Home country of residence____________________

Any known medical conditions: Yes ☐ if yes please specify__________________

No ☐ ____

Do you drink alcohol? Yes ☐ if yes, how many units per week? ________

No ☐ Calculation: strength (ABV) x volume (ml) ÷ 1,000 = units

Do you smoke currently? Yes ☐ No ☐ if yes, how many cigarettes per day ________

Favourite sport: __________________________

Favourite food: __________________________

Average sleep period: < 7 h/day ☐ > 7 h/day ☐

Weekend: Outdoor activity: <2hrs/day ☐ 2-4 hrs/day ☐ >4hrs/day ☐

Indoor screen time TV hrs/day?______ Computer hrs/day?______

Average exercise (or sport) hours per week: ____ hours

Using local gym facilities: Yes ☐ No ☐

Do you believe that you are physically fit: Yes ☐ No ☐

If doing less than 3 hours moderate exercise or activity per week, what is the main reason?

Don't want to ☐ No time ☐ No facility ☐

others (please specify) _________________________
